# Associations of Government-Mandated Closures and Restrictions With Aggregate Mobility Trends and SARS-CoV-2 Infections in Nigeria

**DOI:** 10.1001/jamanetworkopen.2020.32101

**Published:** 2021-01-20

**Authors:** Daniel O. Erim, Gbemisola A. Oke, Akinyele O. Adisa, Oluwakemi Odukoya, Olalekan A. Ayo-Yusuf, Theodora Nawa Erim, Tina N. Tsafa, Martin M. Meremikwu, Israel T. Agaku

**Affiliations:** 1 Parexel International, Durham, North Carolina; 2 Department of Periodontology and Community Dentistry, University of Ibadan, Nigeria; 3 Department of Oral Pathology, University of Ibadan, Nigeria; 4 Department of Community Health and Primary Care, University of Lagos, Nigeria; 5 Sefako Makgatho Health Sciences University, Ga-Rankuwa, South Africa; 6 Holly Hill Hospital, Raleigh, North Carolina; 7 Benue State University, Makurdi, Nigeria; 8 Department of Paediatrics, University of Calabar Teaching Hospital, Calabar, Nigeria; 9 Department of Oral Epidemiology, Harvard Dental School, Boston, Massachusetts

## Abstract

**Importance:**

To prepare for future coronavirus disease 2019 (COVID-19) waves, Nigerian policy makers need insights into community spread of COVID-19 and changes in rates of infection associated with government-mandated closures and restrictions.

**Objectives:**

To measure the association of closures and restrictions with aggregate mobility and the association of mobility with severe acute respiratory syndrome coronavirus 2 (SARS-CoV-2) infections and to characterize community spread of COVID-19.

**Design, Setting, and Participants:**

This cross-sectional study used aggregated anonymized mobility data from smartphone users in Nigeria who opted to provide location history (from a pool of up to 40 million individuals) collected between February 27 and July 21, 2020. The analyzed data included daily counts of confirmed SARS-CoV-2 infections and daily changes in aggregate mobility across 6 categories: retail and recreation, grocery and pharmacy, parks, transit stations, workplaces, and residential. Closures and restrictions were initiated on March 30, 2020, and partially eased on May 4, 2020.

**Main Outcomes and Measures:**

Interrupted time series were used to measure associations of closures and restrictions with aggregate mobility. Negative binomial regression was used to evaluate associations between confirmed SARS-CoV-2 infections and mobility categories. Averted infections were estimated by subtracting cumulative confirmed infections from estimated infections assuming no closures and restrictions.

**Results:**

Closures and restrictions had negative associations with mean change in daily aggregate mobility in retail and recreation (–46.87 [95% CI, –55.98 to –37.76] percentage points; *P* < .001), grocery and pharmacy (–28.95 [95% CI, –40.12 to –17.77] percentage points; *P* < .001), parks (–43.59 [95% CI, –49.89 to –37.30] percentage points; *P* < .001), transit stations (–47.44 [95% CI, –56.70 to –38.19] percentage points; *P* < .001), and workplaces (–53.07 [95% CI, –67.75 to –38.39] percentage points; *P* < .001) and a positive association with mobility in residential areas (24.10 [95% CI, 19.14 to 29.05] percentage points; *P* < .001). Most of these changes reversed after closures and restrictions were partially eased (retail and recreation: 14.63 [95% CI, 10.95 to 18.30] percentage points; *P* < .001; grocery and pharmacy: 15.29 [95% CI, 10.90 to 19.67] percentage points; *P* < .001; parks: 6.48 [95% CI, 3.98 to 8.99] percentage points; *P* < .001; transit stations: 17.93 [95% CI, 14.03 to 21.83] percentage points; *P* < .001; residential: –5.59 [95% CI, –9.08 to –2.09] percentage points; *P* = .002). Additionally, every percentage point increase in aggregate mobility was associated with higher incidences of SARS-CoV-2 infection in residential areas (incidence rate ratio [IRR], 1.03 [95% CI, 1.00 to 1.07]; *P* = .04), transit stations (IRR, 1.02 [95% CI, 1.00 to 1.03]; *P* = .008), and workplaces (IRR, 1.01 [95% CI, 1.00 to 1.02]; *P* = .04). Lastly, closures and restrictions may have been associated with averting up to 5.8 million SARS-CoV-2 infections over the study period.

**Conclusions and Relevance:**

In this cross-sectional study, closures and restrictions had significant associations with aggregate mobility and were associated with decreased SARS-CoV-2 infections. These findings suggest that future anticontagion measures need better infection control and contact tracing in residential areas, transit stations, and workplaces.

## Key Points

**Question:**

Were coronavirus disease 2019 (COVID-19)–related government-mandated closures and restrictions associated with changes in aggregate mobility and the spread of COVID-19 in Nigeria?

**Findings:**

In this cross-sectional study of data from smartphone users throughout Nigeria, closures and restrictions had significant associations with aggregate mobility trends and may have been associated with averting up 5.8 million severe acute respiratory syndrome coronavirus 2 infections over the study period. Accelerated community spread of COVID-19 was noted in residential areas, transit hubs, and workplaces.

**Meaning:**

These findings suggest that government-mandated closures and restrictions may have slowed the spread of COVID-19 in Nigeria.

## Teaser

This cross-sectional study uses data from smartphone users to examine the association of government-mandated closures and restrictions with aggregate mobility and the association of changes in mobility with confirmed SARS-CoV-2 infections in Nigeria.

## Introduction

On January 30, 2020, the director general of the World Health Organization (WHO) declared the coronavirus disease 2019 (COVID-19) outbreak a public health emergency of international concern, the WHO’s highest level of alarm.^1^ All countries were advised or reminded to prepare for containment, active surveillance, early detection, isolation, case management, and contact tracing.^1^ Most countries, including Nigeria (Africa’s largest economy and most populous country), responded accordingly, and part of the Nigerian government’s response included socioeconomic and public health interventions to reduce impacts of the pandemic.^2,3,4^ Socioeconomic interventions included providing cash transfers, lines of credit, and food assistance to poor and high-risk households, while public health interventions included government-mandated closures and restrictions on schools, social gatherings, and all forms of transportation (locally referred to as *lockdown*).^4,5,6^ Closures and restrictions were initiated on March 30, 2020, and partially eased on May 4, 2020. On examining events that occurred before and after closures and restrictions were initiated, some have described Nigeria as having been ill-prepared for the COVID-19 pandemic and have argued that socioeconomic interventions provided weak protections to families at high risk.^4^ Others question benefits of closures and restrictions, considering the potential negative impacts on the Nigerian economy, accompanying widespread starvation, infeasibility of social distancing given common living conditions and cultural norms, increased incidence of crime (including armed robberies, domestic abuse, and gender/sexual violence), and numerous reports of social gatherings.^6,7,8,9,10,11,12,13^ A few studies have shown that reduced aggregate mobility and social isolation reduced the spread of COVID-19 in other countries.^14,15,16,17^ However, little is known about how closures and restrictions were associated with aggregate mobility or spread of COVID-19 in Nigeria. This information may inform future public health responses to subsequent waves of severe acute respiratory syndrome coronavirus 2 (SARS-CoV-2) infections.^18^ To address this gap in knowledge, our study goals were to measure the association of government-mandated closures and restrictions with aggregate mobility, to evaluate associations between aggregate mobility and number of individuals with laboratory confirmed SARS-CoV-2 infections, and to estimate the number of SARS-CoV-2 infections that may have been averted by closures and restrictions.

## Methods

### The Data

We created a data set using information from 2 sources: the Nigeria Center for Disease Control (for data describing daily counts of SARS-CoV-2 infections confirmed via reverse transcription–polymerase chain reaction testing [RT-PCR] between February 27, 2020 [day 1, when the first SARS-CoV-2 infection was confirmed in Nigeria], and July 21, 2020) and COVID-19 Community Mobility Reports from Google (Alphabet) for Nigeria-specific mobility data between February 27 and July 21, 2020.^4,5^ These analyses involved use of unidentifiable publicly available data, and thus qualifies as nonhuman research under the Common Rule (45 CRF 46); therefore, ethical review was not sought. We followed the Strengthening the Reporting of Observational Studies in Epidemiology (STROBE) reporting guideline.

### Measures

#### Individuals With Confirmed SARS-CoV-2 Infections

Data on daily counts of individuals with RT-PCR–confirmed SARS-CoV-2 infections followed a quadratic trend over the study period. During analyses, we specified this variable as a moving mean of daily counts in 5 to 7 days after an index date. The moving mean prevents simultaneity between aggregate mobility and daily counts of confirmed infections, while 5 to 7 days reflects COVID-19’s median incubation period.^19,20,21^

#### Aggregate Mobility and Other Variables of Interest

Records of transportation trends in a given country or state were provided as mobility data by Google COVID-19 Community Mobility Reports.^22^ The reports provide aggregated and anonymized mobile device location data from users with a Google account on their smartphone who opted to have their location data available to Google Location History.^22,23^ These data are provided as a percentage change from baseline activity, in which baseline is defined as the median value for a given day of the week from a 5-week period between January 3 and February 6, 2020.^22,23^ Mobility data are categorized into 6 groups: retail and recreation, grocery and pharmacy, parks, transit stations, workplaces, and residential. *Retail and recreation* refers to areas with restaurants, cafes, shopping centers, theme parks, museums, libraries, and movie theaters. *Grocery and pharmacy* refers to areas with grocery markets, food warehouses, farmers’ markets, specialty food shops, drug stores, and pharmacies. *Parks* refers to areas with local parks, national parks, public beaches, marinas, dog parks, plazas, and public gardens. *Transit stations* refers to areas with public transport hubs, such as bus, taxicab, or train stations. Lastly, *workplaces* and *residential* refers to places of work and residence, respectively. The data do not contain any indication of sample size or characteristics of individuals who contributed to it. Other variables of interest were a time-varying trichotomous indicator of periods before and after closures and restrictions were initiated and partially eased (ie, *closures and restrictions indicator,* set to 0 for February 27 to March 29, 2020; 1 for March 30 to May 3, 2020; or 2 for May 4 to July 21, 2020), duration (ie, the number of days between day 1 and an index date), and duration squared (to match the quadratic trend observed in the data).

### Statistical Analysis

#### Associations of Closures and Restrictions With Aggregate Mobility

We measured associations of closures and restrictions with all mobility categories using interrupted time series (ITS) regression models, which work by specifying outcome trends in preimplementation and postimplementation periods and measuring the vertical distance between proximal ends of both trends and the difference between gradients of both trends (ie, immediate and sustained outcomes associated with an intervention). We used separate ITS models to estimate outcomes associated with initiating and partially easing closures and restrictions. Our null hypothesis was that initiating or partially easing closures and restrictions did not have a significant association with aggregate mobility. We tested our hypotheses using ITS models with the following specification:

*Y_t_* = α + β_1_ × (*day_t_* – *day*_0_) + β_2_ × (*day_t_* – *day*_0_) × (*day_t_* ≥ *day*_0_) + β_3_ × (*day_t_* ≥ *day*_0_) + ε_t_

in which *Y_t_* represents daily changes in aggregate mobility per category; α, the constant term; β_1_, the indicated mobility trend in the preimplementation period; β_2_, the sustained change associated with initiating or partially easing closures and restrictions; β_3_, the immediate change associated with initiating or partially easing closures and restrictions; and ε_t_, the error term at time *t*. We used 2-sided α = .05 as the threshold for statistical significance. We also excluded mobility data for 11 days preceding March 30, 2020, when closures and restrictions were initiated, as observed changes were most likely anticipatory.

#### Associations of Aggregate Mobility With Confirmed SARS-CoV-2 Infections

We used a negative binomial regression model to evaluate mean daily counts of confirmed SARS-CoV-2 infections as a function of mobility categories, the closures and restrictions indicator, duration, and duration squared.^24^ Our null hypothesis was that there was no association between mean daily counts of confirmed infections and any mobility category. Again, we used 2-sided α = .05 as the threshold for statistical significance, and we expected the size and significance of estimated incidence rate ratios (IRRs) per mobility category to provide insights into where the most community spread of SARS-CoV-2 infections were occurring.

#### Estimated Number of SARS-CoV-2 Infections Averted Associated With Closures and Restrictions

Possibly averted infections were estimated as the difference between cumulative expected infections (without closures and restrictions) and cumulative confirmed infections (with closures and restrictions). We estimated daily expected infections (without closures and restrictions) using the method of recycled predictions, a simulation exercise in which restricted mobility data (ie, changes observed between February 27 and March 1, 2020) and a constant closures and restrictions indicator (set to zero) were combined with unaltered duration data (and its squared form) in a Poisson regression model to estimate expected daily counts of infections assuming no closures or restrictions were enacted.^25,26^ We validated this approach by estimating daily counts of confirmed infections (with closures and restrictions) and assessing estimation accuracy objectively using the root mean squared logarithmic errors and subjectively by comparing estimated data with observed data. In sensitivity analyses, we used a negative binomial regression models for estimations. All analyses were performed with Stata statistical software version 13 (StataCorp).

## Results

### Descriptive Statistics

**Figure 1.**
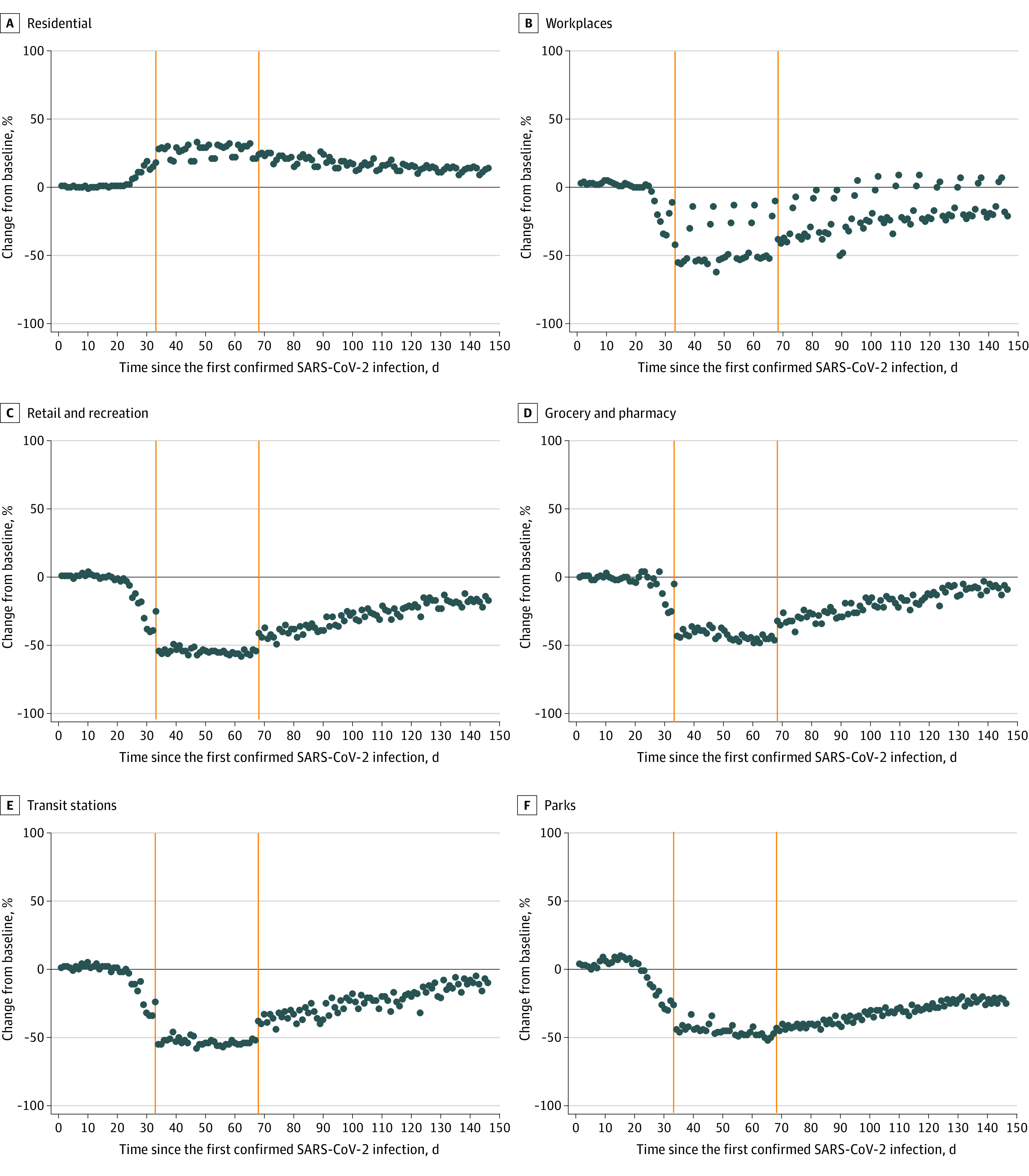
Aggregate Mobility Trends in Nigeria by Area Between February 27 and July 21, 2020 Severe acute respiratory syndrome coronavirus 2 (SARS-CoV-2) infections were confirmed by reverse transcription–polymerase chain reaction testing. Orange lines indicate when closures and restrictions were implemented and then partially eased.

Changes in aggregate mobility before and after closures and restrictions were initiated and partially eased are presented in Table 1 and Figure 1. Mean changes in daily aggregate mobility were minimal before closures and restrictions were initiated, but notably lower afterward in retail and recreation (–53.57 [95% CI, –55.41 to –51.73] percentage points), grocery and pharmacy (–41.29 [95% CI, –43.76 to –38.81] percentage points), parks (–45.46 [95% CI, –47.20 to –43.71] percentage points), transit stations (–52.46 [95% CI, –54.36 to –59.55] percentage points), and workplaces (–42.91 [95% CI, –48.35 to –37.48] percentage points), but not in residential areas (26.66 [95% CI, 25.05 to 28.26] percentage points). Additionally, each week, there were a pair of spikes in aggregate mobility trends for workplace areas after closures and restrictions were initiated, specifically occurring on Saturdays and Sundays (Figure 1B). Lastly, cumulative confirmed SARS-CoV-2 infections increased exponentially after closures and restrictions were initiated.

**Table 1.** Descriptive Statistics

Area	Mean changes in daily aggregate mobility, percentage points (95% CI)		
	Before initiating closures and restrictions (21 d)	Closures and restrictions were in place (35 d)	After partially easing closures and restrictions (79 d)
Retail and recreation	0.52 (–0.19 to 1.24)	–53.57 (–55.41 to –51.73)	–28.04 (–30.13 to –25.95)
Grocery and pharmacy	–0.62 (–1.38 to 0.15)	–41.29 (–43.76 to –38.81)	–18.65 (–20.66 to –16.63)
Parks	4.19 (2.91 to 5.47)	–45.46 (–47.20 to –43.71)	–32.71 (–34.35 to –31.07)
Transit	1.38 (0.57 to 2.19)	–52.46 (–54.36 to –59.55)	–23.38 (–25.57 to –21.19)
Workplaces	2.33 (1.65 to 3.01)	–42.91 (–48.35 to –37.48)	–19.04 (–22.30 to –15.78)
Residential	0.48 (0.20 to 0.75)	26.66 (25.05 to 28.26)	16.67 (15.73 to 17.62)
Individuals with confirmed SARS-CoV-2 infection, No.	6	2554	37 782

Abbreviation: SARS-CoV-2, severe acute respiratory syndrome.

### Association of Closures and Restrictions With Aggregate Mobility

Immediate changes associated of initiating closures and restrictions were significant decreases in aggregate mobility trends for retail and recreation (–46.87 [95% CI, –55.98 to –37.76] percentage points; *P* < .001), grocery and pharmacy (–28.95 [95% CI, –40.12 to –17.77] percentage points; *P* < .001), parks (–43.59 [95% CI, –49.89 to –37.30] percentage points; *P* < .001), transit stations (–47.44 [95% CI, –56.70 to –38.19] percentage points; *P* < .001), and workplaces (–53.07 [95% CI, –67.75 to –38.39] percentage points; *P* < .001) and a significant increase in the aggregate mobility trend for residential areas (24.10 [95% CI, 19.14 to 29.05] percentage points; *P* < .001]). The sustained change associated with initiating closures and restrictions was a significant decrease in the aggregate mobility trend for parks (–0.54 [95% CI, –0.78 to –0.30] percentage points per day; *P* < .001) (Table 2).

**Table 2.** Associations of Government-Mandated Closures and Restrictions With Aggregate Mobility Trends in Nigeria During the COVID-19 Pandemic

Mobility domains	Initiating closures and restriction (duration = 56 d)					Easing closures and restrictions (duration = 114 d)				
	Immediate change		Sustained change		*R*^2^	Immediate change		Sustained change		*R*^2^
	Regression coefficient, percentage points (95% CI)	*P* values	Regression coefficient, percentage points per d (95% CI)	*P* value		Regression coefficient, percentage points (95% CI)	*P* values	Regression coefficient, percentage points per d (95% CI)	*P* value	
Retail and recreation	–46.87 (–55.98 to –37.76)	<.001	–0.08 (–0.36 to 0.21)	.60	0.98	14.63 (10.95 to 18.30)	<.001	0.58 (0.32 to 0.84)	<.001	0.92
Grocery and pharmacy	–28.95 (–40.12 to –17.77)	<.001	–0.23 (–0.57 to 0.11)	.19	0.94	15.29 (10.90 to 19.67)	<.001	0.72 (0.40 to 1.04)	<.001	0.88
Parks	–43.59 (–49.89 to –37.30)	<.001	–0.54 (–0.78 to –0.30)	<.001	0.98	6.48 (3.98 to 8.99)	<.001	0.61 (0.44 to 0.78)	<.001	0.89
Transit stations	–47.44 (–56.70 to –38.19)	<.001	–0.13 (–0.43 to 0.16)	.37	0.98	17.93 (14.03 to 21.83)	<.001	0.58 (0.31 to 0.85)	<.001	0.91
Workplaces	–53.07 (–67.75 to –38.39)	<.001	0.48 (–0.04 to 1.02)	.07	0.77	6.67 (–6.04 to 19.36)	.30	–0.05 (–0.57 to 0.47)	.84	0.45
Residential	24.10 (19.14 to 29.05)	<.001	0.04 (–0.13 to 0.21)	.65	0.93	–5.59 (–9.08 to –2.09)	.002	–0.21 (–0.37 to –0.04)	.01	0.72

Immediate changes associated with partially easing closures and restrictions were significant increases in aggregate mobility trends for retail and recreation (14.63 [95% CI, 10.95 to 18.30] percentage points; *P* < .001), grocery and pharmacy (15.29 [95% CI, 10.90 to 19.67] percentage points; *P* < .001), parks (6.48 [95% CI, 3.98 to 8.99] percentage points; *P* < .001), and transit stations (17.93 [95% CI, 14.03 to 21.83] percentage points; *P* < .001) and a significant decrease in the aggregate mobility trend for residential areas (–5.59 [95% CI, –9.08 to –2.09] percentage points; *P* = .002). Sustained changes associated with partially easing closures and restrictions were significant increases in aggregate mobility trends for retail and recreation (0.58 [95% CI, 0.32 to 0.84] percentage points per day; *P* < .001), grocery and pharmacy (0.72 [95% CI, 0.40 to 1.04] percentage points per day; *P* < .001), parks (0.61 [95% CI, 0.44 to 0.78] percentage points per day; *P* < .001), and transit (0.58 [95% CI, 0.31 to 0.85] percentage points per day; *P* < .001) and a significant decrease in the mobility trend for residential areas (–0.21 [95% CI, –0.37 to –0.04] percentage points per day; *P* = .01) (Table 2 and Figure 1).

### Associations Between Aggregate Mobility Measures and Confirmed SARS-CoV-2 Infections

There were significant increases in incidence rates for every percentage point increase in aggregate mobility across residential areas (IRR, 1.03 [95% CI, 1.00 to 1.07]; *P* = .04), transit stations (IRR, 1.02 [95% CI, 1.00 to 1.03]; *P* = .008), and workplaces (IRR, 1.01 [95% CI, 1.00 to 1.02]; *P* = .04) (Table 3).

**Table 3.** Associations of Aggregate Mobility and Confirmed Severe Acute Respiratory Syndrome Coronavirus 2 Infections

Key independent variable	Incidence rate ratios (95% CI)^a^	*P* value
Retail and recreation	0.99 (0.97-1.02)	.56
Grocery and pharmacy	1.00 (0.99-1.02)	.96
Parks	0.99 (0.97-1.01)	.19
Transit stations	1.02 (1.00-1.03)	.008
Workplaces	1.01 (1.00-1.02)	.04
Residential	1.03 (1.00-1.07)	.04

^a^ 95% CIs were estimated using robust SEs. Pseudo *R*^2^ = 0.30.

### Estimated SARS-CoV-2 Infections Averted Associated With Closures and Restrictions

**Figure 2.**
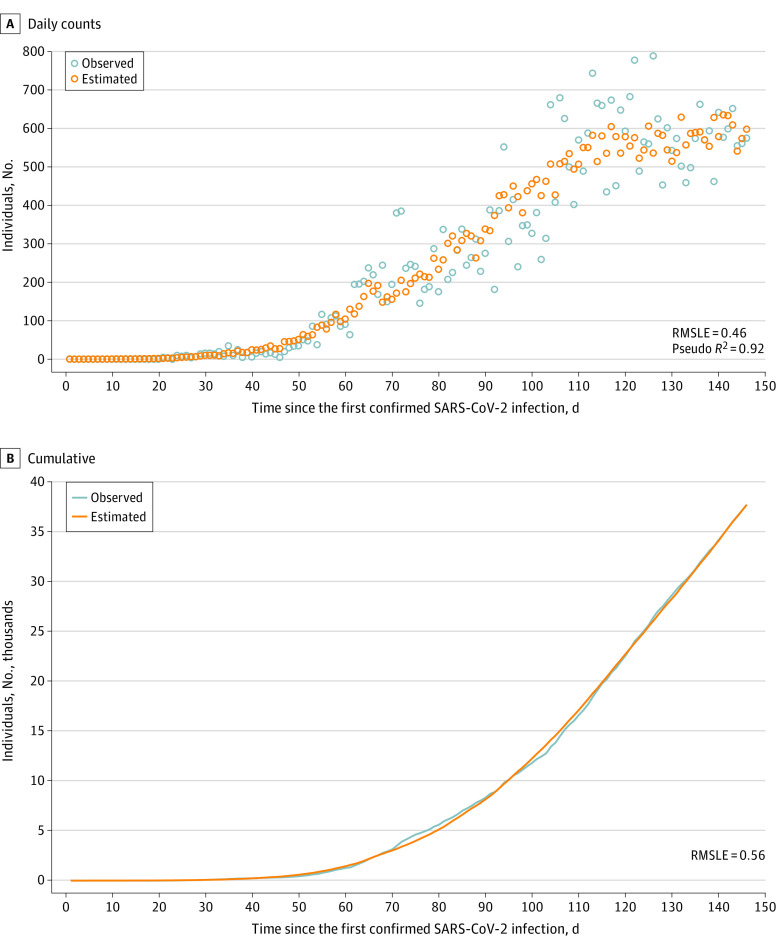
Observed and Estimated Daily and Cumulative Confirmed Severe Acute Respiratory Syndrome Coronavirus 2 (SARS-CoV-2) Infections in Nigeria SARS-CoV-2 infections were confirmed by reverse transcription–polymerase chain reaction testing. Cumulative infections were obtained by progressively summing daily observed and estimated infections. RMSLE indicates root mean squared logarithmic error.

Results from the validation exercise indicate that the observed and estimated number of individuals with SARS-CoV-2 infection over the study period (daily and cumulative) were similar and root mean squared logarithmic errors were low (Figure 2). At the end of the period, 37 782 individuals had RT-PCR–confirmed SARS-CoV-2 infections in Nigeria. The Poisson model estimated that there would have been 5 780 161 individuals with SARS-CoV-2 infections if closures and restrictions had not been initiated and if everything else remained the same. Hence, an estimated 5 742 379 averted infections may have been associated with initiating closures and restrictions.

## Discussion

The findings of this cross-sectional study suggest that the closures and restrictions in Nigeria were associated with significant changes in mobility trends and may have been associated with averting approximately 5.8 million SARS-CoV-2 infections, which equates to approximately 3.0% of the total population of Nigeria, over the study period. Although there is limited literature on the number of averted SARS-CoV-2 infections associated with anticontagion policies in other countries, a study by Hsiang and colleagues^27^ provided estimates for Italy (2.1 million [3.5% of the total population]), Iran (4.9 million [6.0% of the total population]), United States (4.8 million [1.5% of the total population]), South Korea (11.5 million [22.3% of the total population]), and China (37 million [2.7% of the total population]). These countries may have been selected based on data availability and implementation of partial or total government-mandated closures. Nonetheless, although Hsiang and colleagues^27^ used a different study design and approach, their findings are largely consistent with ours. We also found evidence that community spread of COVID-19 was significantly higher in residential areas, transit stations, and workplace areas. These findings are consistent with the literature and have several implications for public health research, policy, programs, and practice.^14,15,16,17^

Our findings support the use of restricted mobility as a measure for infection control in Nigeria should there be additional COVID-19 waves in the future. However, as restrictions on movement are unsustainable in the long term, future closures and restrictions, if warranted, need to be more effective. Suggested areas for improvement include tougher restrictions on movement and more robust contact tracing in residential areas, transit hubs and workplaces; greater testing capacity and more political support for testing; greater access to COVID-19 data for policy and process evaluation to identify opportunities for efficiency gains; and more personal responsibility above and beyond the public health campaign dubbed *the 3 Ws* (ie, washing hands [or using hand sanitizer] regularly, wearing a cloth mask over the nose and mouth, and waiting 6 feet apart [or social distancing]).^28^

Regarding personal responsibility, the current director of the Nigeria Center for Disease Control in a recent interview stated “[Nigeria is] a very liberal society… [and does not have] a government that controls. That makes it difficult when you do need government control and enforcement to make people do things that are not comfortable to do: Wear masks, not gather, keep distance from each other. The other thing is cultural. We are a people’s country; we talk, we laugh, we joke, we dance, we love music, sports, and all those things that bring people together. And all of a sudden, we can’t do any of those.”^28^ Despite restrictions on movement and social gatherings, we observed spikes in mobility trends in workplaces on Saturdays and Sundays. Anecdotal evidence suggests that these spikes are most likely owing to public gatherings for social and religious events on indicated days, and these emphasize a need for greater personal responsibility to control the spread of COVID-19.

### Strengths and Limitations

This study has several limitations. There may have been residual confounding from failing to control for changes in SARS-CoV-2 testing capacity or prevalence of the 3 Ws (ie, the health belief model): these omitted variables probably increased over time, may be associated with number of confirmed SARS-CoV-2 infections, and may bias our estimated IRRs.^28,29^ However, these biases are likely to be minimal, as indicated omitted variables are expected to be correlated with the closures and restrictions indicator.^28^ There is limited information on the accuracy of labels for mobility categories. Anecdotal evidence suggests that categories may overlap with one another (eg, residential areas within or close to workplaces and transit stations*)*. Such mislabeling may result in type 2 error on IRRs. There were no suitable controls with which to compare Nigeria’s data, including synthetic controls, as most countries had voluntary or government-mandated closures and restrictions (either partial or total) or some other anticontagion measure. In estimating the association of closures and restrictions with aggregate mobility trends, we excluded changes in mobility data that were probably anticipatory. However, on including these previously excluded data, our findings remained robust. Specific aspects of closures and restrictions that were not directly assessed were travel bans on visits to or from 13 countries starting March 18, 2020 (day 21), on all international flights starting March 23, 2020 (day 26), and on domestic flights starting April 20, 2020 (day 54). Additionally, not all states initiated closures and restrictions at the same time: they were initiated in Abuja, Lagos, and Ogun on March 30, 2020 (day 33), and in Bauchi and all other states on April 2, 2020 (day 36). However, our findings suggest that staggered travel bans and initiation of closures and restrictions may not have been associated with significant changes in aggregate mobility.^14,15^ Similarly, national-level data from Google’s COVID-19 Community Mobility Reports^22^ may only capture information from a selection of states. For example, while national-level mobility data had no missing observations over the study period, approximately 30% of state-level mobility data were missing. Google has stated that gaps may occur when mobility data fail to meet its quality and privacy standards.^22^ There may be a pattern to missing observations in state-level mobility data, and little is known about how these missing observations were handled while national-level mobility data were being aggregated. Furthermore, missing state-level information may impede generalizability of our findings to affected areas (eg, states with lower smartphone ownership). In the same vein, mobility data were obtained from internet-connected smartphones, and this may introduce noncoverage bias, as smartphone owners are less likely to reside in rural areas. Additionally, we analyzed data on confirmed SARS-CoV-2 infections in Nigeria; data on untested or unconfirmed infections were unavailable. While we presume that estimated expected infections (ie, assuming no closures and restrictions) would have also been tested and confirmed, this may not be the case, given limitations in Nigeria’s testing capacity.^4,28^ Similarly, owing to scarcity of reliable mortality data, we were unable to measure the association of closures and restrictions with COVID-19 deaths.

## Conclusions

This cross-sectional study found that government-mandated closures and restrictions in Nigeria owing to COVID-19 had significant associations with aggregate mobility and may have been associated with averting up to 5.8 million SARS-CoV-2 infections. Additionally, community spread of COVID-19 in Nigeria may have been faster in residential areas, transit stations, and workplaces.
